# GDF15, EGF, and Neopterin in Assessing Progression of Pediatric Chronic Kidney Disease Using Artificial Intelligence Tools—A Pilot Study

**DOI:** 10.3390/ijms26052344

**Published:** 2025-03-06

**Authors:** Kinga Musiał, Jakub Stojanowski, Agnieszka Bargenda-Lange, Tomasz Gołębiowski

**Affiliations:** 1 Department of Pediatric Nephrology, Wrocław Medical University, Borowska 213, 50-556 Wrocław, Poland; 2 Department of Nephrology and Transplantation Medicine, Wrocław Medical University, Borowska 213, 50-556 Wrocław, Poland

**Keywords:** artificial intelligence, chronic inflammation, epidermal growth factor, growth differentiation factor 15, leave-one-out cross-validation (LOOCV) method, multilayer perceptron (MLP) network model

## Abstract

Cell-mediated immunity and chronic inflammation are hallmarks of chronic kidney disease (CKD). Growth differentiation factor 15 (GDF15) is a marker of inflammation and an integrative signal in stress conditions. Epidermal growth factor (EGF) is a tubule-specific protein that modulates the regeneration of injured renal tubules. Neopterin is a product of activated monocytes and macrophages and serves as a marker of cell-mediated immunity. Our aim was to assess the role of the above-mentioned parameters in the progression of CKD in children using artificial intelligence tools. The study group consisted of 151 children with CKD stages 1–5. EGF, GDF15, and neopterin serum concentrations were assessed by ELISA. The patients’ anthropometric data, biochemical parameters, EGF, GDF15, and neopterin serum values were implemented into the artificial neural network (ANN). The most precise model contained EGF, GDF15, and neopterin as input parameters and classified patients into either CKD 1–3 or CKD 4–5 groups with an excellent accuracy of 96.77%. The presented AI model, with serum concentrations of EGF, GDF15, and neopterin as input parameters, may serve as a useful predictor of CKD progression. It suggests the essential role of inflammatory processes in the renal function decline in the course of CKD in children.

## 1. Introduction

The progression of chronic kidney disease (CKD) is a compound process where uremic toxicity influences immunocompetent cells, leading to subclinical inflammation. Cell-mediated immunity, in concert with growth factors, triggers damage to tubular cells and subsequent fibrosis of tubulointerstitial tissue, which is responsible for the irreversible character of the disease.

Growth differentiation factor 15 (GDF15), a member of transforming growth factor (TGF)-β superfamily, is barely detectable in all tissues except for the placenta [1]. However, as a stress-induced cytokine, it increases under unfavorable conditions, and this fact has established GDF15 as a marker related to inflammation, tissue injury, metabolic disturbances, or tumorigenesis [2,3]. This pluripotency may also result from the downstream regulation of multiple signaling pathways [4]. However, in clinical practice, the major focus is still on the role of GDF15 in vascular inflammation, atherosclerosis, hypertension, and cardiac remodeling [4]. Over recent years, this interest has surpassed the cardiovascular system and expanded to the respiratory tract, kidneys, and systemic diseases like diabetes [5]. Thus, GDF15 elevation in adults with CKD was first associated with an increased rate of heart failure and higher overall mortality rate, and then the connection with CKD progression was revealed [6,7]. Limited data from pediatric studies confirmed the presence of increased plasma/serum GDF15 concentrations in children with CKD without features of cardiovascular disease [8,9]. The latter suggested that GDF15’s early rise in the course of CKD in children marks cell damage and inflammation rather than cardiovascular disease.

Epidermal growth factor (EGF) is a distal tubule-specific protein, mediating renal repair after injury through its receptor (EGFR) [10,11]. The latter undergoes activation by various stimuli, including GDF15, and is then responsible for the downstream regulation of several signaling pathways [12]. Despite its abilities to promote proliferation, differentiation, and survival, data concerning serum EGF in nephrology are limited. Contrary to GDF15, EGF presents heat stability, enriching the spectrum of markers specific for renal tubules and adding to the compound analysis of adaptive mechanisms against progressive renal dysfunction in the course of CKD. So far, our previous analysis of EGF serum concentrations in CKD children confirmed their decreased values in patients on chronic dialysis [9].

Neopterin, a pyrazino-pyrimidine compound, originates from monocytes and macrophages stimulated by T-cell driven interferon (IFN) gamma. Thus, it serves as a marker of cell-mediated immunity in various conditions, such as autoimmunity, cancer, diabetes, and viral infections, including COVID-19 [13,14,15,16]. Such a vast field of activities mimics that of GDF15 and may suggest the choice of neopterin as a universal marker of altered immune reactions. Immune deficits accompanying CKD would then justify the use of neopterin as a marker of cell-mediated inflammation. Predictably, elevated serum neopterin concentrations correlated with CKD severity in adult patients [17,18]. Our investigation revealed increased serum neopterin values in children with advanced stages of CKD and on chronic dialysis [19].

Those markers were never analyzed regarding their functional connections and reciprocal influence on signaling pathways in the context of the CKD spectrum (stages 1–5 treated conservatively). The concomitant evaluation of GDF15, EGF, and neopterin throughout declining renal function could reveal the potential role of chronic inflammation in CKD progression in its pediatric specificity. Moreover, we decided to enhance the power of our calculations by using machine learning methods and to create a model with the best distinctive properties of early (1–3) vs. advanced (4–5) stages of CKD.

Therefore, the aim of study was to assess the serum concentrations of GDF15, EGF, and neopterin in children with CKD on conservative treatment, to confront them with classical biochemical indicators of CKD-related complications, and to verify the usefulness of new markers in distinguishing between early and advanced stages of CKD using artificial intelligence tools.

## 2. Results

### 2.1. GDF15, EGF, and Neopterin Serum Concentrations

**Figure 1:**
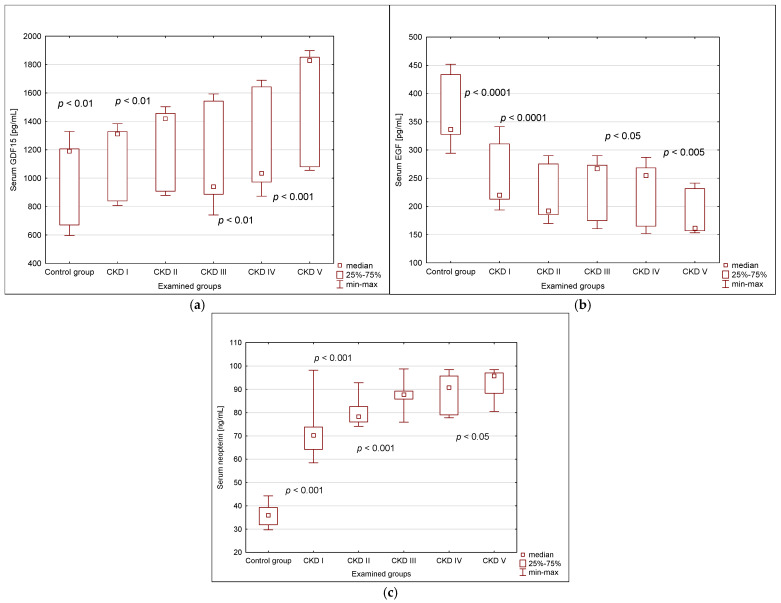
Parameter serum concentrations in examined groups: (**a**) GDF15; (**b**) EGF; (**c**) neopterin; CKD I–V—chronic kidney disease stage I–V; GDF15—growth differentiation factor 15; EGF—epidermal growth factor.

The EGF serum concentrations decreased gradually, whereas the GDF15 and neopterin values rose systematically with CKD progression, keeping statistically significant inter-stage differences (Figure 1).

### 2.2. Correlations Between GDF15, EGF, Neopterin Serum Concentrations, and Classical Markers of CKD

The EGF, GDF15, and neopterin values correlated with each other, and the strongest relation was present between EGF and GDF15 (R = 0.94, *p* = 0.000001). The analyzed parameters also correlated with clinical markers of CKD-related complications and with the advancement of CKD (Table 1).

However, when the statistical significance of selected markers vs. CKD progression was tested on linear regression analysis, their predictive abilities in the evaluation of CKD staging were variable (Table 2).

### 2.3. Multilayer Perceptron (MLP) Network Modeling

**Figure 2:**
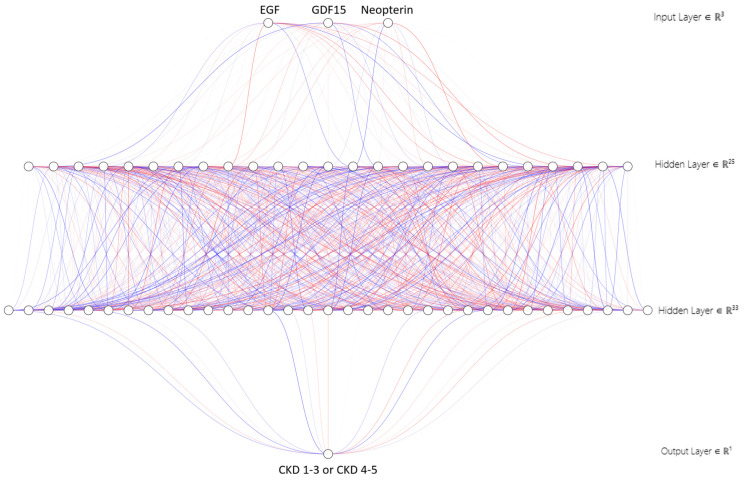
The artificial neural network model with EGF, GDF15, and neopterin as input data. The colors of the connections between the neurons represented by the round nodes correspond to the weights of the connections—positive weight values are more intensely red, and negative weights are more intensely blue. The input layer contains 3 input parameters, then there are 2 hidden layers, in which each of the neurons of a given layer has connections with all of the next layer. The output layer is one neuron, which takes the value 0 or 1 corresponding to CKD 1–3 or CKD 4–5.

The best 3-parameter model, assessed using the MCC on the testing set, contained EGF, GDF15, and neopterin as input parameters and classified patients into either CKD stages 1–3 or CKD stages 4–5 groups (Figure 2).

The selection based on choosing the highest MCC value on the training set enabled the choice of parameters best classifying the patients into groups with early (CKD stages 1–3) and advanced (CKD stages 4–5) chronic kidney disease, based on the mentioned parameters. At the same time, both groups could be differentiated not only on the basis of individual parameters but also in a complex way using a combination of all three. This proves that a constellation of results can contribute to better prediction and differentiation than a single parameter.

This model puts new patients into appropriate classes with an excellent accuracy of 96.77%. The value of the area under the receiver operator curve (ROC) for the model on the training set was 0.9510. Once the training set was selected based on the model resistant to overfitting, further validation of the testing set was performed with the use of leave-one-out cross-validation (LOOCV) due to its better performance on small datasets. The value of the area under the ROC curve for the testing set was 0.9375, while the average value based on the LOOCV method for the testing set was 0.9225.

**Figure 3:**
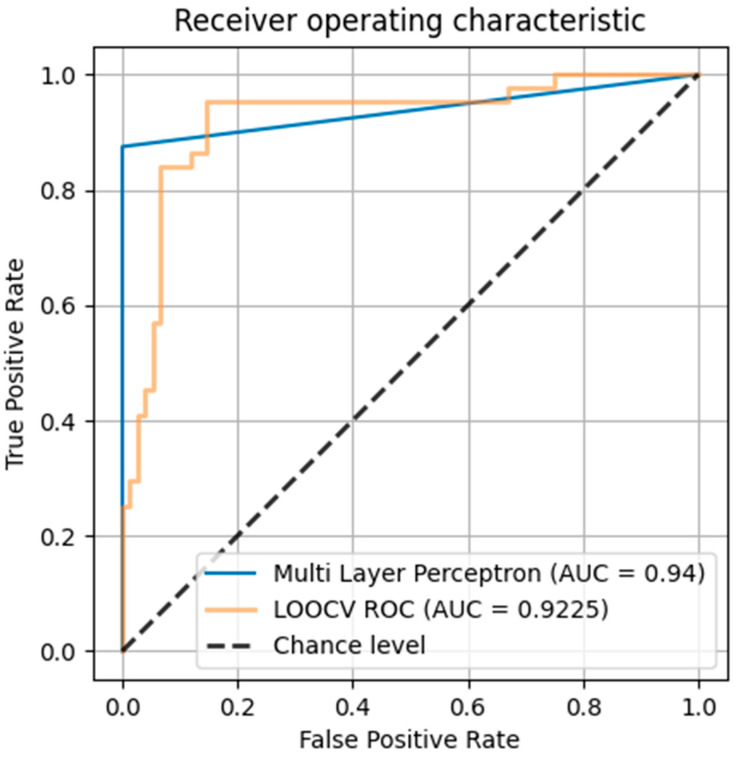
ROC for multilayer perceptron model. AUC—area under the curve, LOOCV—leave-one-out cross-validation.

The positive predictive ability of the model was 0.9691, and the sensitivity was 0.9677. Regarding individual endpoints, in the testing set, the model detected CKD stages 1–3 with a positive predictive value (PPV) of 0.96 and 100% sensitivity and CKD stages 4–5 with an excellent PPV of 1.00 and 88% sensitivity. In the testing set, the proportion of records with a CKD 1–3 endpoint accounted for 23 of 31 records, or 74%. It is estimated that this variability may result from the difference in size of both classes in the training and testing sets (Figure 3).

The Matthews correlation coefficient (MCC) value was 0.9157, suggesting that despite some imbalance in the input set, the model is ultimately able to effectively classify patient outcome records into the appropriate endpoint categories (CKD stages 1–3 or CKD stages 4–5).

The approach relative to other models proved the MLP’s superiority. The XGBoost model, based on the clinical parameters (incl. albumin, PTH, uric acid, and hemoglobin), achieved an accuracy of 96.77% and MCC of 0.9221, which means it performed slightly better in the confusion matrix, but the selection method did not take into account the markers we tested. The random forest model, based on sex, height, GDF15, and neopterin concentrations, achieved identical results to the analyzed multilayer perceptron network but required over 100 component trees to achieve an accuracy of 96.77% and MCC of 0.9157.

## 3. Discussion

Chronic inflammation localized within the kidney, leading to irreversible damage and fibrosis, is one of the hallmarks of CKD, adding to the progression of renal function decline. Meanwhile, cell-mediated altered responses to toxemia, hypoxia, pathogens, etc., together with immune deficits, may aggravate/multiply comorbidities responsible for the CKD patient’s outcome, transforming the local pathology into systemic disease.

The most convincing data on local-to-global transition concerned cardiovascular (CV) complications in adults with CKD. Namely, the increased GDF15 serum concentrations, known originally as markers of mortality in patients with isolated cardiovascular problems, turned into useful indices of CV condition in patients with concomitant kidney damage. Moreover, serum/plasma GDF15 concentrations predicted renal impairment in the course of diabetic nephropathy and incidence of chronic kidney disease (CKD) in the general population [20,21], whereas elevated serum GDF15 increased the risk of CKD progression [7]. Additionally, a strong correlation between the circulating level of GDF15 and its intrarenal expression was confirmed [7].

Our results on serum GDF15 concentrations, increasing with declining eGFR values, seem to confirm previous findings on CKD progression in adults with elevated GDF15. Moreover, with the use of AI tools, the proof of GDF15’s ability to distinguish, in constellation with EGF and neopterin, between early and advanced stages of CKD was available. With LOOCV, all the models containing at least three input parameters were tested, and those with GDF15, EGF, and neopterin surpassed those based on parameters like anthropometric data, classical markers of inflammation, blood cell count, and calcium phosphate metabolism. Our results underline the paramount, yet underestimated, role of chronic inflammation in CKD pathophysiology among children. The fact that such correlation was found in the pediatric CKD population, where cardiovascular complications are already present, but not mortal yet, could strengthen the convincing power of this finding. Therefore, elevated GDF15 may be of predictive value for CKD progression even before CV issues take the lead.

On the other hand, the pluripotency of GDF15 as a marker, being considered a diagnostic/prognostic index of obesity, cancer development, and response to chemotherapy, suggests the common pathway of action/activation with other molecules [2,5,22]. Indeed, as mentioned previously, GDF15 is one of the activators of EGFR [12].

Surprisingly, contrary to multiple results concerning GDF15’s role in various pathologies, research on circulating EGF is rather scarce. The major focus was on tubule-specific urinary EGF, which has proven its utility as a surrogate marker of tubular regenerative potential after acute kidney injury [23]. However, the recent cohort studies gave conflicting results regarding urine EGF association with incident CKD in adults [24,25], whereas the decreased urinary EGF concentrations, together with increased KIM-1 and NGAL, were identified among markers connected with fast adult CKD progression [26]. Likewise, renal function decline in CKD children was associated with low levels of EGF in urine [27,28]. Our previous analysis confirmed decreased serum EGF concentrations in children on chronic dialysis [9], and that observation remains in line with current findings of EGF’s gradual decline along with aggravating CKD. Moreover, this tendency may at least partly explain the decreasing urinary content of EGF in CKD patients. EGF, as a low molecular mass (6 kDa) protein, is easily filtered through the intact glomerular filtration barrier and may well undergo massive leakage throughout the damaged one. Therefore, future investigation is needed to prove the potential connection between serum EGF and urine EGF. Until such results appear, the decreased serum EGF concentrations may be treated as a surrogate marker of CKD-related immune dysfunction.

Among the three tested molecules, neopterin seemed to be the most appropriate candidate for the assessment of cell-mediated immunity. Its direct role in monocyte–macrophage transition, the clue process to inflammation-triggered damage of the kidney, favored the meaning of circulating neopterin as a marker of intensity of monocyte migration and in situ transition in the course of CKD. Surprisingly, as in the case of GDF15, neopterin has been used rather for the prediction of disease severity and outcome in heart failure patients [29]. Moreover, previous analysis suggested neopterin’s usefulness in the prediction of cardiovascular and all-cause mortality in patients after kidney transplantation, denying its association with renal outcome [30]. However, other studies suggested correlations between elevated serum neopterin concentrations and CKD severity in adult patients [17,18]. Our results confirm those findings, additionally showing the dynamics of neopterin rise along with CKD progression in the pediatric population.

Yet the combined role of serum GDF15, EGF, and neopterin in differentiating between early and advanced stages of CKD, as well as its advantage over classical markers of CKD progression, was only revealed by the machine learning application. Classical statistical methods gave general suggestions on possible connections between the three tested parameters and their potential towards distinction between early and advanced CKD, but it was the MLP network that revealed their superiority over classical CKD markers used in everyday clinical practice.

Modeling using artificial intelligence allows the identification of risk factors and significant correlations that are elusive to classical analysis. Since a model based on selected input parameters accurately classifies sets of input data into appropriate categories, it directly means that, on the basis of these parameters and the relationships that constitute the core of the model, it is possible to draw conclusions about the factors leading to a given state. No doubt, the mathematical model only interprets numbers, and the task of the scientists using AI is to give them practical meaning and real dimension.

As a result, any translation of the results of AI analysis into practice has a natural limitation, which is the clinical meaning of the data. In this particular case, MLP enabled confirmation of the connections between analyzed molecules that could be primarily deduced from molecular functional links between them. MLP’s advantage over other tested models was due to its readability and also its potential for further development with new data. The model established in this study has underlined the impact that disturbances in cell-mediated immunity, such as inflammation and monocyte–macrophage transition, have on the progression of CKD in children. The advantage of this model over other known hallmarks of chronic kidney damage raises the question of whether to include inflammatory markers in the panel of CKD markers. In the era of point-of-care testing, when panels of markers serve better than single parameter analysis, such an option of an inflammatory CKD set is worth considering. However, in order to verify this hypothesis, further observational studies are needed.

We also have to acknowledge the limitations of the study. First, the groups of patients were not numerous, so the AI methods had to be adjusted to the small dataset. The shortcoming of the lack of external validation was the natural consequence of the low number of patients originating from one clinical center. This was a retrospective cross-sectional study, so we could not observe the progression of CKD in particular patients over time. The analysis took into account a few classical parameters of inflammation and selected growth factors, so we did not analyze the full spectrum of complex cell-mediated inflammatory conditions in the course of chronic kidney disease.

Taking into account the clinical background of the analyzed material, we concluded that, based on serum GDF15, EGF, and neopterin concentrations, the groups of children with CKD stages 1–3 could be differentiated from patients with CKD stages 4–5. Consequently, this means that these biochemical parameters may be jointly associated with the progression of CKD in the pediatric population, although these conclusions require verification in the course of future investigation, performed on a larger dataset, involving external validation.

## 4. Materials and Methods

### 4.1. Basic Characteristics

The study group consisted of 151 children with pre-dialysis CKD stages 1–5 and 25 age-matched children with nocturnal enuresis and normal kidney function, who served as controls. Basic anthropometric and biochemical data of the studied groups are given in Table 3 and Table 4.

Blood samples were drawn from peripheral veins during routine control analyses after an overnight fast. The samples were clotted for 30 min, centrifuged at 4 °C, 1000× *g* for 15 min, and then the serum was stored at −80 °C until assayed.

### 4.2. Assay Characteristics

The EGF, GDF15, and neopterin serum concentrations were assessed by ELISA (EGF–R&D Systems (Abingdon, UK), reagent kit DEG00; GDF15–R&D Systems (Abingdon, UK), reagent kit DGD150; the neopterin—Tecan Group Ltd. (Männedorf, Switzerland), reagent kit RE59321). The standards and serum samples were transferred to 96-well microplates pre-coated with recombinant antibodies to human EGF, GDF15, and neopterin. Measurements were performed according to the manufacturer’s instructions, and the results were calculated by reference to standard curves. The serum biochemistry parameters were measured using automated routine diagnostic tests on the Beckman Coulter AU2700 analyzer. The eGFR values were calculated according to the Schwartz formula [31].

### 4.3. Classical Statistical Analysis

The results were expressed as median values and interquartile ranges. Since the null hypothesis of normality of distribution was rejected by the Shapiro–Wilk test, comparisons were evaluated by using nonparametric tests (Kruskal–Wallis, Mann–Whitney U). The relations between parameters were assessed by Spearman’s correlation coefficient and by linear regression analysis. The linear regression equations were calculated as y = βx + a (y—dependent variable, β—regression coefficient, x—independent variable, a—constant term). We presented only those equations where both the regression coefficient and constant term were statistically significant. Statistical analysis was performed using Statistica ver. 13.5 (StatSoft, Tulsa, OK, USA). A *p* value < 0.05 was considered significant.

### 4.4. Database Analysis by Multilayer Perceptron (MLP) Network

The anonymized patient database was implemented into the artificial neural network. Anthropometric data, biochemical parameters, EGF, GDF15, and neopterin serum concentrations were included in the model. The serum creatinine and eGFR values, as direct classifiers of CKD stage, were excluded.

The original database containing 151 patient records was randomly divided into a training set and a test set in a ratio of 80:20. Based on the data from the training set, all statistically possible models containing at least three input parameters were built. The minimum number of three input variables was set due to the impossibility of creating a model with less than three input variables in our program. The recursion method allowed us to explore all possible combinations of input data containing no less than three unique input variables. In order to determine the metavariables of the neural network, i.e., the number of neurons in a given hidden layer, the brute force method was used. For this purpose, two loops nested within each other were used. The minimum number of neurons in each hidden layer was set to one. The maximum number of neurons in any of the two layers was arbitrarily set to no more than 50. Each additional neuron is responsible for increasing the complexity of the neural network. Our goal was to simplify the model as much as possible in order to perform visual interpretation and avoid overfitting. Overfitting is the phenomenon of a model being too strong a fit to the training data, which prevents effective functioning on new input data, including that from the testing set. Complex models tend to remember data and not necessarily find patterns. Machine learning is most concerned with finding patterns and rules that enable data classification, especially forecasting.

Each model was evaluated using the AUROC value. The model with the best AUROC score was run on the test set to evaluate the model’s behavior on completely new data and evaluated using the LOOCV value [32].

Nested iterations involve starting a loop within a loop, i.e., each time one loop runs, another one is started, the execution of which allows moving to the next step in the parent program loop. In terms of computational complexity, this is the least effective solution, but due to the relatively small number of operations performed internally and using a fast computer, many models are checked, and the best one can be chosen. Different numbers of neurons in two layers of the neural network were assessed, as well as the parameter initiating the formation of the neural network, the so-called seed or state. Each model was assessed based on the AUROC parameter result, and the best model was assessed using the leave-one-out cross-validation (LOOCV) method, which is preferred for assessing performance on a small data set [33].

The LOOCV is a procedure used to estimate the performance of machine learning algorithms when they are used to make predictions on data not used to train the model. LOOCV is a variant of cross-validation in which the set is checked on subsets resulting from the elimination of a single element. If the validated set consists of k elements, this method provides k estimates of a model’s performance on the dataset.

The Matthews correlation coefficient (MCC) is a classification model parameter that becomes closer to 1.0 as the overall model performance improves in all four fields of the confusion matrix. The more true and fewer false classifications there are, the higher the MCC value the model achieves. The way MCC calculates the model ensures insensitivity to class size imbalances for differentiation. This parameter allows for the actual assessment of data that contains more representatives of the selected group. Thus, even an inefficient classification model can achieve high accuracy by entering a larger group of labels [34,35].

The entire program, including the user interface, was developed by the authors of the manuscript using the Python (3.12.9) programming language with available libraries in accordance with their licenses.

## 5. Conclusions

The presented model of an artificial neural network, with serum concentrations of EGF, GDF15, and neopterin as input parameters, shows potential towards the prediction of CKD progression in the pediatric population. Our results obtained in the course of this pilot study require further verification on a larger dataset, with external validation based on a group of patients from another center. Nevertheless, the promising current outcome suggests the essential role of inflammatory processes, defined by newly discovered markers, in the renal function decline in the course of CKD in children.

## Figures and Tables

**Table 1:** Correlations between analyzed parameters and clinical markers of chronic kidney disease (CKD) in children; eGFR—estimated glomerular filtration rate; CRP—C-reactive protein.

Parameters	eGFR [mL/min/1.73 m^2^]	Uric Acid [mg/dL]	Albumin [g/dL]	Hemoglobin [g/dL]	Parathormone [pg/mL]	CKD Stage
EGF	R = 0.37 *p* = 0.00001	R = −0.19 *p* = 0.024	R = 0.49 *p* = 0.00001	R = 0.29 *p* = 0.0004	R = −0.39 *p* = 0.000004	R = −0.42 *p* = 0.000001
GDF15	R = −0.36 *p* = 0.00001	R = 0.18 *p* = 0.029	R = −0.47 *p* = 0.00001	R = −0.26 *p* = 0.001	R = 0.39 *p* = 0.000004	R = 0.43 *p* = 0.000001
Neopterin	R = −0.51 *p* = 0.0000001	R = 0.26 *p* = 0.003	R = −0.09 *p* = 0.34	R = −0.38 *p* = 0.000007	R = 0.24 *p* = 0.008	R = 0.58 *p* = 0.0000001

**Table 2:** The linear regression analysis of predictive abilities of analyzed parameters in children with CKD.

Dependent Variable	Independent Variable	Regression Coefficient β	Constant Term	Coefficient of Determination R^2^	*p*
Serum EGF	Serum GDF15	−0.97	397.57	0.91	0.000001
	CKD stage	−0.7	339.11	0.12	0.0009
Serum GDF15	CKD stage	0.53	145.68	0.69	0.007
Serum neopterin	CKD stage	0.74	5.48	0.24	0.02
CKD stage	Serum neopterin	0.54	0.07	0.39	0.000001

**Table 3:** Basic characteristics of the examined groups of children with chronic kidney disease (CKD stages 1–5) and the control group. F—female, M—male.

Examined Groups	Number of Patients	Gender F M	Age [Years] Median Values (Lower–Upper Quartile)	BMI [kg/m^2^] Median Values (Lower–Upper Quartile)
CKD 1	26	9	12.7	17.7
		17	(8.4–14.1)	(16.9–20.3)
CKD 2	25	9	9.5	16.5
		16	(5.1–13.4)	(15.5–18.4)
CKD 3	51	19	11.1	16.5
		21	(7.3–14.9)	(14.7–19.7)
CKD 4	28	14	10.9	15.8
		14	(9.9–14.5)	(15.0–19.3)
CKD 5	21	10	11.6	17.2
		11	(8.1–14.4)	(15.2–19.0)
Control group	25	15	10.3	18.2
		10	(5.9–15.2)	(16.1–21.0)

**Table 4:** Selected laboratory results of children with chronic kidney disease (CKD), presented as median values and values of lower and upper quartiles. eGFR—estimated glomerular filtration rate; CRP—C-reactive protein.

CKD Stage	eGFR [mL/min/1.73 m^2^]	CRP [ng/L]	Albumin [g/dL]	Hemoglobin [g/dL]	Parathormone [pg/mL]
1	114	0.29	4.4	13.5	29.5
	(110–135)	(0.13–0.96)	(4.2–4.7)	(12.9–14.7)	(23.2–42.4)
2	74	0.33	4.5	12.9	63.0
	(70–81)	(0.22–1.22)	(4.1–4.6)	(11.8–13.7)	(30.2–88.4)
3	45	0.35	4.4	12.6	84.1
	(36–51)	(0.20–0.62)	(4.2–4.6)	(11.3–13.5)	(59.0–120.0)
4	23	0.21	4.4	11.8	190.8
	(19–27)	(0.14–0.73)	(3.8–4.7)	(10.4–12.3)	(126.9–344.3)
5	10	0.36	4.3	10.5	296.9
	(8–12)	(0.16–0.82)	(3.7–4.5)	(9.0–12.0)	(190.6–456.5)

## Data Availability

The datasets generated and analyzed during the current study are available from the corresponding author upon reasonable request.
